# Improved neonatal outcomes by multidisciplinary simulation—a contemporary practice in the demonstration area of China

**DOI:** 10.3389/fped.2023.1138633

**Published:** 2023-06-08

**Authors:** Chenguang Xu, Qianshen Zhang, Yin Xue, Chun-Bong Chow, Chunxiao Dong, Qian Xie, Po-Yin Cheung

**Affiliations:** ^1^ NICU, University of Hong Kong-Shenzhen Hospital, Shenzhen, China; ^2^ Child Health Department, Longhua District Maternal & Child Healthcare Hospital, Shenzhen, China; ^3^ Department of Obstetrics, The University of Hong Kong-Shenzhen Hospital, Shenzhen, China; ^4^ Centre for the Studies of Asphyxia and Resuscitation, Neonatal Research Unit, Royal Alexandra Hospital, University of Alberta, Edmonton, AB, Canada; ^5^ NICU, University of Alberta, Edmonton, AB, Canada

**Keywords:** neonatal resuscitation, in-situ simulation training, multidisciplinary, neonatal outcomes, asphyxia

## Abstract

**Background:**

Simulation-based training improves neonatal resuscitation and decreases perinatal mortality in low- and middle-income countries. Interdisciplinary in-situ simulation may promote quality care in neonatal resuscitation. However, there is limited information regarding the effect of multidisciplinary in-situ simulation training (MIST) on neonatal outcomes. We aimed to investigate the impact of MIST on neonatal resuscitation in reducing the incidence of neonatal asphyxia and related morbidities.

**Methods:**

Weekly MIST on neonatal resuscitation has been conducted through neonatal and obstetrical collaboration at the University of Hong Kong-Shenzhen Hospital, China, since 2019. Each simulation was facilitated by two instructors and performed by three health care providers from obstetric and neonatal intensive care units, followed by a debriefing of the participants and several designated observers. The incidence of neonatal asphyxia, severe asphyxia, hypoxic-ischemic encephalopathy (HIE), and meconium aspiration syndrome (MAS) before (2017–2018) and after (2019–2020) the commencement of weekly MIST were analyzed.

**Results:**

There were 81 simulation cases including the resuscitation of preterm neonates of different gestational ages, perinatal distress, meconium-stained amniotic fluid, and congenital heart disease with 1,503 participant counts (225 active participants). The respective incidence of neonatal asphyxia, severe asphyxia, HIE, and MAS decreased significantly after MIST (0.64%, 0.06%, 0.01%, and 0.09% vs. 0.84%, 0.14%, 0.10%, and 0.19%, respectively, all *P* < 0.05).

**Conclusions:**

Weekly MIST on neonatal resuscitation decreased the incidence of neonatal asphyxia, severe asphyxia, HIE, and MAS. Implementation of regular resuscitation simulation training is feasible and may improve the quality of neonatal resuscitation with better neonatal outcomes in low- and middle-income countries.

## 1. Introduction

Most neonatal deaths, which contribute to almost half of the deaths of children under 5 years old, occur in low- and middle-income countries and regions, with preterm birth complications and intrapartum-related events (previously called birth asphyxia) as the most common causes (1). Neonatal resuscitation has the potential to prevent perinatal deaths of nearly 2 million infants due to asphyxia every year (2). Effective and prompt resuscitation in the delivery room could improve neonatal outcomes (3–7). However, the basic skills of neonatal resuscitation in the delivery room and compliance with guidelines are insufficient (8, 9).

Many studies support the efficacy of neonatal resuscitation training in improving knowledge and performance (10), through the translation of the science of resuscitation into a training program. Neonatal resuscitation training resulted in significant improvement in clinical outcomes, with decreased neonatal and perinatal mortality in low- and middle-income countries (10–13). After the Neonatal Resuscitation Program (NRP®) training was implemented in China, the incidence of neonatal asphyxia in respective regions declined from 6.32% in 2003 to 2.94% in 2008, and the mortality rate declined from 7.55 to 3.41 per 10,000 livebirths (14).

While resuscitation training was found to improve immediate knowledge and skill acquisition, one-time training may not be sufficient for sustained knowledge, or the incorporation of key skills related to resuscitation into clinical practice (15). In addition to that, improved performance in the simulation environment may not be transferable to the clinical setting (16). Skills decline more than knowledge over time (17, 18) and structured practice and refresher training could maintain neonatal resuscitation skills (18, 19). In 2015, the ILCOR pointed out that the increase in training frequency could improve providers' self-confidence and recommended that the training frequency should be more than once a year (20). However, evidence remains inconclusive on the maintenance of resuscitation skills, the optimal interval of repeated training, and conversion to clinical performance. As defined by the Society for Simulation in Healthcare in the Healthcare Simulation Dictionary (second edition), in-situ simulation is the training taking place in the actual patient care setting/environment in an effort to achieve a high level of fidelity and realism (21). In-situ simulation-based training can foster and maintain newborn ventilation skills in a multidisciplinary delivery unit staff in a high-resource setting (22). Multidisciplinary in-situ simulation training (MIST) may effectively improve technical skills and teamwork in neonatal resuscitation. However, the impact of real-life neonatal resuscitation is unknown (23).

We describe our 2-year practice of collaborative MIST on neonatal resuscitation and concomitant neonatal outcomes. The goal of MIST on neonatal resuscitation was primarily to strengthen behavioral skills, and secondarily to reinforce technical skills and the adherence to NRP® algorithm through simulation and subsequent debriefing. We aimed to evaluate the impact of MIST conducted through neonatal and obstetrical collaboration on the incidence of neonatal asphyxia and related complications. The feasibility and barriers to developing MIST on neonatal resuscitation were also investigated and discussed. We tested the hypothesis that frequent and regular MIST on neonatal resuscitation would improve acute neonatal outcomes.

## 2. Methods

This study was approved by the Institutional Review Board of the University of Hong Kong-Shenzhen Hospital (HKU-SZH), China. Shenzhen is a relatively high-income region in an area known as the Demonstration Area of China, representing a developed area in a developing country where innovative practices can be tried and implemented. The HKU-SZH has approximately 6,000–8,000 deliveries annually and a neonatal intensive care unit (NICU) with 40 level-III and level-II beds. The NICU has an average admission of 350 high-risk neonates per year. Since its opening in 2012, HKU-SZH has introduced evidence-based neonatal resuscitation workshops to provide training to NICU staff and midwives. The curriculum is based on ILCOR guidelines and NRP® with modifications for the context in China (24). In HKU-SZH, all neonatologists, NICU physicians and nurses, midwives, and obstetricians have to satisfactorily complete a 2-day neonatal resuscitation workshop and successful recertification every 2 years.

**Figure 1:**
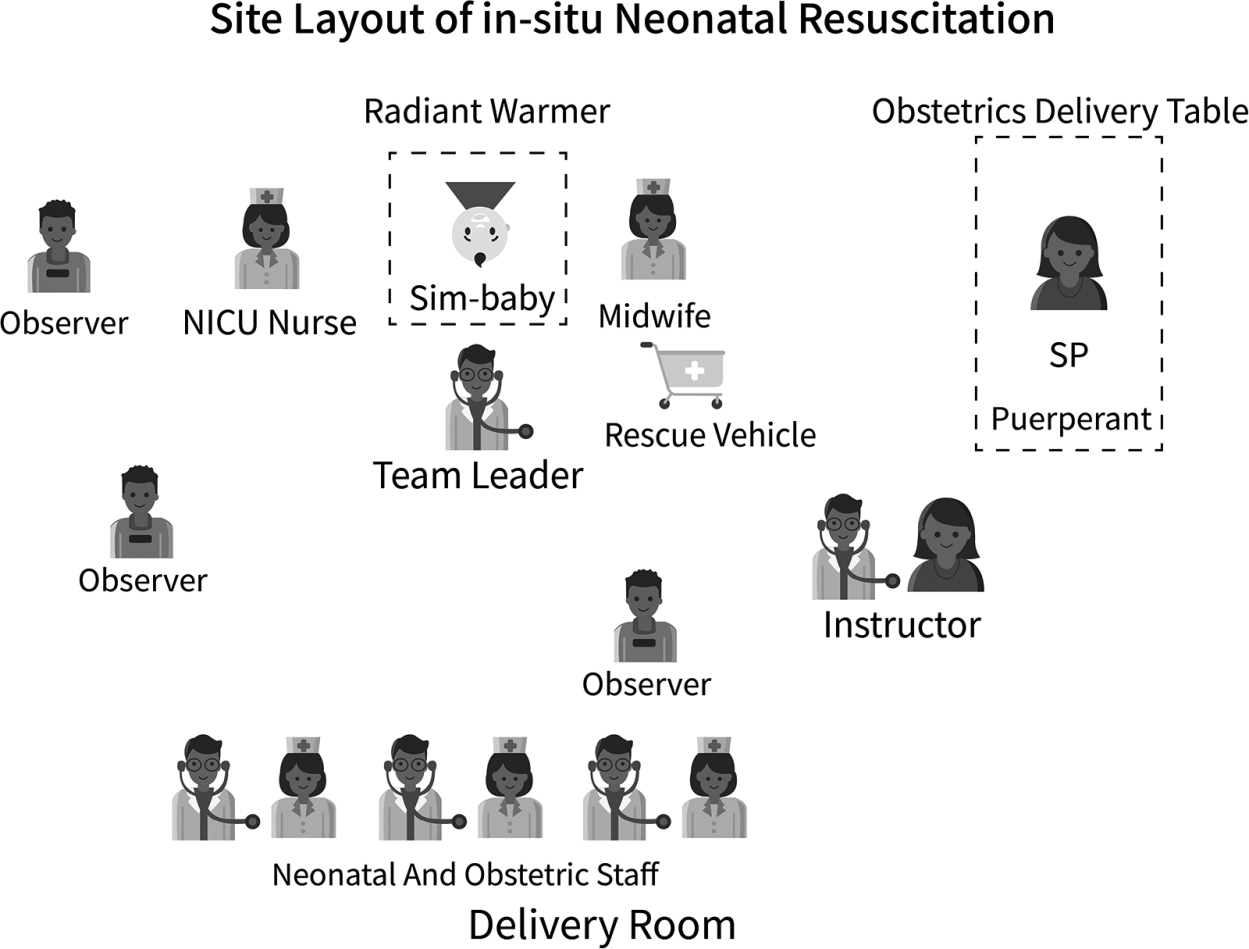
Site layout of in-situ neonatal resuscitation in HKU-SZH. The simulation was set in the delivery room or other clinical environment. Resuscitation providers included a NICU physician (usually as the leader), midwife, NICU nurse, and NICU second-line doctor according to the situation or if providers require additional help. Designated observers usually involved a NICU physician, midwife, and NICU nurse. Others included NICU and obstetric staff, trainees. SP, standardized patient.

Since 2019, MIST on neonatal resuscitation has been conducted through neonatal and obstetrical collaboration regularly every Wednesday morning in a delivery room. After completion of training in neonatal resuscitation, all providers would participate in the weekly MIST. The simulation was facilitated by 2 instructors, participated by 3 providers from the obstetric unit and NICU, followed by debriefing which was observed by several designated observers (Figure 1). The set-up of the resuscitation cart, which is used for simulation only, is the same as that for clinical use. The instructors were certified locally by experienced Canadian and American NRP® certified instructors through HKU-SZH Train-the-Trainer programs after they completed an instructor training course in China, as currently there is no mutual agreement between the American Academy of Pediatrics and the China Medical Association on NRP® training.

The Supplementary Material shows the different phases of the MIST and the roles of instructors, providers, and designated observers.

### 2.1. Different Phases of MIST

#### 2.1.1. Briefing

Every simulation began with learning objectives including both technical and non-technical skills and discussion about an event that was yet to happen or a hypothetical case. The technical skills included correct techniques for specific procedures and MR SOPA ventilation corrective steps (Mask adjustment, Reposition of the airway, Suction the mouth and nose, Opening mouth, increasing Pressure, and placing Alternative airway) or identification of some indications for critical steps of resuscitation. Non-technical skills mainly referred to teamwork and communication.

#### 2.1.2. Resuscitation simulation

The team leader was supposed to identify the risk factors for resuscitation, using the checklist to prepare and assign a clear task for each team member. The practitioners performed simulated resuscitation on a neonatal manikin according to scenarios based on the 7th edition of the NRP textbook and NRP® 2015 algorithm in a real clinical environment (6, 20). Low-fidelity manikin (Laerdal® Newborn Anne) was used from January 2019 to May 2020, and high-fidelity manikin (Laerdal® SimNewB™) was used from June 2020 to December 2020. Instructors, designated observers, and other staff would not disrupt nor discuss during the progression of resuscitation. Video recording was not taken during the simulation.

#### 2.1.3. Debriefing

The debriefing was facilitated by instructors immediately after the simulation on-site in the delivery room/postpartum ward/emergency room. If it was a simulation on neonatal resuscitation and stabilization during transport, debriefing was carried out in NICU after transportation was completed. Providers, designated observers, and other staff expressed what was done well and what needed to be improved. Evidenced-based management of the case, compliance with the NRP® guidelines, and teamwork were emphasized.

#### 2.1.4. Conclusions

The key points were summarized by the instructors to ensure that the learning objectives were achieved.

### 2.2. Data collection

Hospital records and NICU clinical database were retrospectively reviewed from a January, 2017 to 31 December, 2020. We compared the incidence of neonatal asphyxia, severe asphyxia, hypoxic-ischemic encephalopathy (HIE), and meconium aspiration syndrome (MAS) before (2017–2018, epoch 1) and after (2019–2020, epoch 2) the commencement of weekly MIST. Asphyxia, low Apgar score, and severe asphyxia were diagnosed according to Chinese national guidelines (25). Asphyxia was defined as Apgar score ≤7 at 1 min or 5 min after birth, and umbilical arterial pH < 7.2. Low Apgar score was defined as Apgar score ≤7 at 1 min or 5 min, whereas umbilical arterial pH ≥ 7.2. Severe asphyxia was defined as an Apgar score ≤3 at 1 min or ≤5 at 5 min after birth, and umbilical arterial pH < 7.0. The diagnosis of HIE was based on the history of perinatal asphyxia and the presence of neurologic dysfunction (26). MAS was diagnosed as respiratory distress in newborns born through meconium-stained amniotic fluid at birth, which could not be explained by other causes (27). The definition of hypothermia was based on that of the WHO, with a rectal temperature of less than 36.5 °C upon admission to NICU (28). These diagnoses were mandated to be entered and checked for accuracy in the clinical database as per hospital and government policies.

Training records of weekly MIST were prospectively collected and those from 1 January, 2019 to 31 December, 2020 were reviewed in this study. We summarize and analyze the categories and teaching objectives of the cases from clinical work, and describe the training process, advantages, and limitations of this training mode.

The primary outcome was the rate of asphyxia, asphyxia or low Apgar score, severe asphyxia, and related complications including HIE and MAS. We also described the characteristics of simulation cases and participants. The characteristics of neonates with MAS were further analyzed and compared between epoch 1 and epoch 2. In the HKU-SZH, we have adopted NRP® recommendation against routine endotracheal intubation and suction since 2017, whereas the guidelines in China continue to recommend routine laryngoscopy with intubation for endotracheal suction for non-vigorous neonates born through meconium-stained amniotic fluid (29). We were therefore interested in examining the incidence of MAS. Due to the large quantity of data and limitations in our medical database, missing information also precluded us from studying other populations in detail including asphyxia, HIE, and hypothermia.

### 2.3. Statistical analyses

The data were presented in mean ± SD or percentage (*n*). Differences between epochs were analyzed by Student's *t*-test for continuous parametric variables and Chi-square test or Fisher Exact test for small sample size as appropriate for categorical variables. SPSS Statistics (v.26) was used for data analyses. A *P* value less than 0.05 was considered statistically significant.

## 3. Results

### 3.1. The weekly MIST on neonatal resuscitation

**Figure 2:**
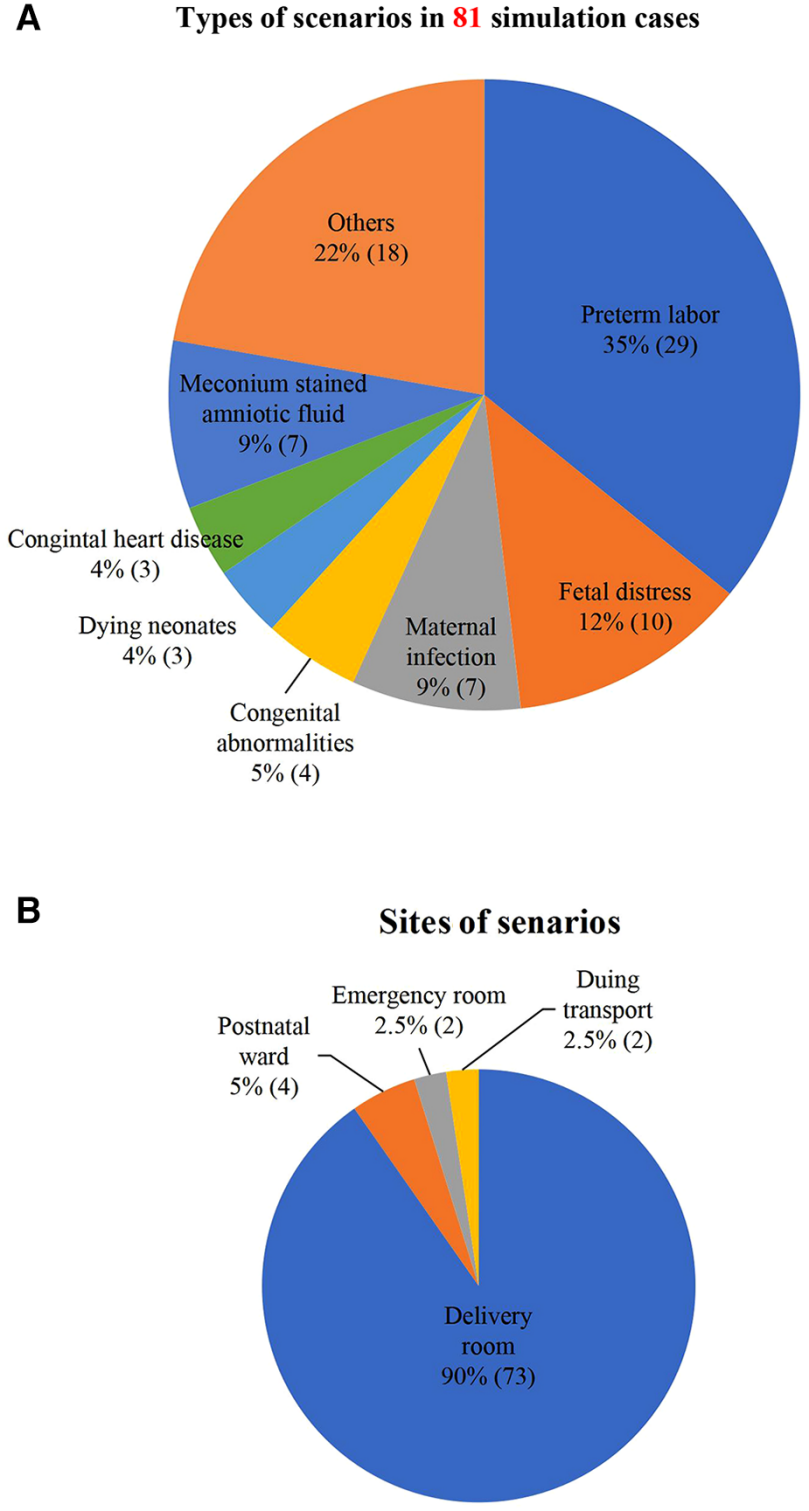
(**A**) Types of scenarios in 81 simulation cases. Congenital malformation included diaphragmatic hernia, digestive tract malformation; Maternal infection included acute chorioamnionitis, maternal HIV infection, maternal suspected COVID-19 infection; Others: aspiration, parents difficult to communicate with, antepartum hemorrhage, insufficient resources (manpower and equipment), maternal complications (myasthenia gravis, epilepsy), pleural effusion, pneumothorax, etc. Data are presented in % (*n*). (**B**) Sites of 81 scenarios. Data are presented in % (*n*).

From 2019 to 2020, there were 81 sessions with simulation cases including resuscitation of preterm neonates with different gestational ages (*n* = 29, 35.8%), fetal distress (*n* = 10, 12.3%), maternal infection (*n* = 7, 8.6%), meconium-stained amniotic fluid (*n* = 7, 8.6%), and congenital abnormalities (*n* = 4, 4.9%) (Figure 2A). Among these sessions, 90.1% happened in the delivery room, and 9.9% in other sites including the emergency room, postpartum ward, and transport vehicle (Figure 2B).

There was an attendance of 1,503 participant-counts (225 active participants) in 81 simulations. The attendance included 38.5% (578) NICU physicians with working experience of more than 3 years, 21.4% (322) NICU nurses, 20.5% (308) medical trainees (NICU working experience less than 3 years or interns), 19.3% (290) midwives, and 0.3% (5) obstetricians. There were 18.6 ± 4.3 participants in each simulation. In each simulation, there were 3.3 ± 0.7 resuscitation providers, and 15.3 ± 4.3 other participants including instructors, designated observers, and other health care providers.

### 3.2. Acute neonatal outcomes during epoch 1 and epoch 2

During the study period, 29,759 live neonates were born in HKU-SZH (15,911 in epoch 1 and 13,848 in epoch 2). Of these live births, 8,313 (52.2%) boys and 7,598 (47.8%) girls were born in epoch 1, and 7,294 (52.7%) boys and 6,554 (47.3%) girls were born in epoch 2. Of these, 1,328 were premature infants (702 in epoch 1 and 626 in epoch 2). There was an increase in forceps deliveries of 3.7% and 8.1% in epochs 1 and 2, respectively (*P* < 0.001), with no significant differences in the rate of cesarean sections and vacuum deliveries. After MIST, there were significant decreases in the incidence of neonatal asphyxia and severe asphyxia (0.64% and 0.06% vs. 0.84% and 0.14% in epoch 1, *P* = 0.045 and *P* = 0.029; respectively), but not for the incidence of neonatal asphyxia or low Apgar score (Table 1). The incidence of asphyxia or low Apgar score in premature infants decreased from 6.27% [44/702] to 3.99% [25/626] but did not reach statistical significance (*P* = 0.062). The incidence of severe asphyxia in premature infants decreased to 0.16% [1/626] [vs. 1.14% (8/702) in epoch 1, *P* = 0.041]. The incidence of HIE and MAS also decreased to 0.01% and 0.09% (vs. 0.10% and 0.19% in epoch 1, *P* = 0.003 and *P* = 0.014; respectively) (Table 1).

**Table 1:** Acute neonatal outcomes of two epochs.

	Epoch 1 (*N* = 15,911)	Epoch 2 (*N* = 13,848)	*P* value
	(Jan 2017–Dec 2018)	(Jan 2019–Dec 2020)	
Neonatal asphyxia or low Apgar score	0.97% (154)	0.8% (111)	0.128
Neonatal asphyxia	0.84% (133)	0.64% (88)	0.045
Low Apgar score	0.13% (21)	0.17% (23)	0.445
Severe asphyxia	0.138% (22)	0.058% (8)	0.029
HIE	0.10% (16)	0.01% (2)	0.003
MAS	0.19% (31)	0.09% (12)	0.014

HIE, hypoxic-ischemic encephalopathy; MAS, meconium aspiration syndrome (based on all live births).

Data were presented as % (*n*). Chi-square test or Fisher Exact test was used in statistical analyses.

We further compared the characteristics of 43 neonates who were diagnosed with MAS in both epochs. There was no difference in birth weight, gestational age, gender, Apgar scores, and delivery room management. Endotracheal suctioning of meconium-stained amniotic fluid was performed in 3.2% (1/31) before and 8.3% (1/12) after MIST (*P* = 0.49). Hypothermia (defined as rectal temperature <36.5°C) at NICU admission was found in 32.3% (10/31) and 8.3% (1/12) in epoch 1 and epoch 2, respectively (*P* = 0.14). The mean body temperature at admission was significantly higher in neonates with MAS born in epoch 2 than that in epoch 1 (37.2 ± 0.7 vs. 36.6 ± 0.8°C, respectively, *P* = 0.022). The proportion of neonates who required respiratory support for epochs 1 and 2 were 51.6% and 83.3% respectively, with non-invasive support in most neonates. There was no difference in the rates of persistent pulmonary hypertension of newborns and pneumothorax (Table 2).

**Table 2:** Comparison of patients with MAS in two epochs.

	Epoch 1 (*N* = 31)	Epoch 2 (*N* = 12)	*P* value
	(Jan 2017–Dec 2018)	(Jan 2019–Dec 2020)	
Birth weight (g)	3,261 ± 429	3,375 ± 344	0.417^a^
Gestational age (w)	40.3 ± 1.0	40.2 ± 0.8	0.91^a^
Male	71.0% (22)	41.7% (5)	0.092^b^
Apgar score at 1 min	6.7 ± 1.7	7.0 ± 2.5	0.70^a^
Apgar score at 5 min	8.0 ± 1.5	8.3 ± 1.2	0.56^a^
Apgar score at 10 min	9.0 ± 0.9	9.1 ± 0.8	0.71^a^
Endotracheal suction	3.2% (1)	8.3% (1)	0.49^b^
Positive pressure ventilation	77.4% (24)	50% (6)	0.137^b^
Chest compressions	9.7% (3)	0% (0)	0.548^b^
Temperature at admission (°C)	36.6 ± 0.79	37.2 ± 0.66	0.022^a^
Hypothermia at admission	32.3% (10)	8.3% (1)	0.14^b^
Invasive ventilation	12.9% (4)	16.7% (2)	1.0^b^
All respiratory support	51.6% (16)	83.3% (10)	0.085^b^
Duration of ventilatory support (days)	1.8 ± 2.6	1.9 ± 1.2	0.87^a^
Persistent pulmonary hypertension of newborn	5% (2)	0% (0)	1.0^b^
Pneumothorax^c^	24% (6 of 25)	9.1% (1 of 11)	0.652^b^

Data were presented as mean ± SD or % (*n*) and analyzed by.

^a^ Student's *t*-test or.

^b^ Chi-square test or Fisher Exact test, respectively.

^c^ Incomplete denominator due to missed data.

## 4. Discussion

We reported our experience in MIST on neonatal resuscitation training in China. In this single-center study, we observed that weekly MIST was feasible and sustainable in a busy tertiary hospital and was also effective in improving acute neonatal outcomes. The 81 simulation cases in epoch 2 were extracted and modified from clinical scenarios. We believe that the mutual translation of knowledge in simulated resuscitation and clinical scenario is an optimal way to improve neonatal resuscitation training and to affect clinical outcomes.

Each simulation training was scheduled at a convenient time regularly and conducted with specific learning objectives. Preterm delivery simulation was one of the most common scenarios in the training program, accounting for one-third of all scenarios. Temperature management, lung protective, and neuroprotective strategies were reinforced in the sessions according to NRP® recommendations. Moreover, we also focused on specific objectives and individualized patient-specific preparations according to different causes of preterm labor such as early administration of antibiotics for preterm neonates with maternal chorioamnionitis. Briefing, simulation, and debriefing are integrated into the process of team training.

Simulation is an effective tool to facilitate the acquisition and maintenance of cognitive, technical, and behavioral skills to deliver safe, effective, and efficient care to neonates (30). While simulation has no risk or threat to patients, the threat to participants has been minimized. All participants acknowledged the cultural concept that errors could occur frequently during resuscitation, and everyone should learn from mistakes (31). We noticed that at the initial stage of our program, participants were reserved and passive in the debriefing session. Once the cultural concept was adopted and a safe learning environment was ensured, participants became more willing to express and share their feelings and opinions. With a multidirectional flow of information and interactive discussion, debriefing is a critical component of the learning process with the aim of improving individual and team performance. We believe that the most important part of the learning process is neither high technology nor excessive time, but a structured method to address what occurred or not occurred in due course (32). Repeated practice may not be adequate without a feedback mechanism (33). During our debriefing practice, the feedback about subsequent management in NICU and follow-up were also updated to the obstetric team for quality improvement and interdisciplinary collaboration. In order to enhance participation and engagement, several designated observers watched the full simulation exercise to provide details of performance and behavioral skills. To our knowledge, there was no previous study identifying the effect of observers. We speculate that the feedback from designated observers may further promote mutual understanding among participants with different working backgrounds and experiences. The provider may be an observer in the subsequent simulation. Role transformation of participants could strengthen mutual understanding and enhance team cooperation.

The majority of participants were passive observers who did not benefit from actual hands-on practice in simulation-based training. During debriefing, the providers and designated observers were encouraged to express opinions first, followed by that of other participants who might also participate in the discussion and raise questions, including the reflection on their own experience and performance in the clinical situation and previous simulation scenarios. Indeed, there were some learners who did not participate in previous neonatal resuscitation workshops, making it impractical to be providers in MIST. These observers might follow the pedagogy framework of “Learn-See-Practice-Prove-Do-Maintain” (LSPPDM) and participate in a neonatal resuscitation workshop for hands-on skill training afterwards, and then participate in another MIST session as providers (34). This would be interesting if this helps the learning process, improve teamwork and retain skills.

The reduced incidence of asphyxia after weekly MIST is interesting and may be multifactorial. These factors included the sustained improvement in resuscitation skills (14), anticipation of perinatal risk and deployment of team members with appropriate skillsets based on risks assessment (35, 36), collaborative and cooperative work in risks assessment and resuscitation between NICU, midwifery and obstetrical staff (37). Interestingly, one observational study in Tanzania showed that frequent, brief (3–5 min weekly) on-site “Helping Babies Breathe” simulation training was associated with a 40% reduction in neonatal mortality at 24 h, which was attributed to the early initiation of basic steps of resuscitation (38). In our simulation scenarios without obvious risk factors, the midwife was set as the initial leader who was expected to perform timely initial resuscitation and effective PPV.

Furthermore, repeated practice helps maintain neonatal resuscitation performance, benefits teamwork behaviors, and improves skill retention (39–41). Indeed, it has been recommended that training should be repeated more frequently than once per year (42). Basic resuscitation skills and special considerations such as preterm delivery have been repeatedly emphasized during simulations. “Delayed cord clamping without delaying resuscitation” and early initiation of CPAP support were focused on in the 29 preterm delivery simulations. Specifically, during the simulation, our providers implemented initial resuscitation steps as appropriate to stimulate/facilitate/support spontaneous respirations while 30s to 60s was timed for (delayed) cord clamping. We believed that these strategies were related to the decline of asphyxia in premature infants.

Neonatal resuscitation is dynamic, complex, and challenging and requires interdisciplinary teamwork and effective communication under intense time pressure and psychomental stress. Complex tasks can lead to deviations from the NRP® algorithm and poor patient outcomes (43). Leadership, teamwork, and effective communication are key components of team performance (44). Clinical drills may help staff be better prepared and resuscitation areas fully equipped (45) with improved communication (46). We aimed to use team-simulated resuscitation to identify system problems, which may hinder the team's ability to perform resuscitation effectively. Multidisciplinary simulation training may promote the transfer of skills into the real-life setting (47), teamwork, team participation, and performance in resuscitation which is associated with improvement in neonatal care (48, 49).

Resuscitation training can happen in a simulation center or *in situ*. Simulation centers may not always be available or practical in low- and middle-income areas. In-situ simulation has the advantages of low-cost, increasing sense of realism, identifying latent system threats and potential risks, increasing participation of staff, and addressing specific issues in the institution (23, 50). In-situ simulation does not only refer to a clinical environment, but should also be integrated with the staff who work there with accessible information and technology (49). The scenarios were therefore designed based on real clinical events and according to institutional team composition.

Although participants might be more satisfied and confident with high-fidelity manikins, the overall performance in teamwork and integrated skills station had little advantages over the low-fidelity manikins (51). The model of MIST described in this report was effective and could be performed in resource-limited settings without high technology. Of note, there were many participants in our study, with an average of 18 in each simulation, including 3 providers and 15 healthcare professionals observing the scenario. The number of participants might have increased stress levels and reduced the training effect. With sufficient time and space, video-assisted recording and broadcasting from the resuscitation room to another separate room where observers are located may be useful in future training.

### 4.1. Challenges

There were a few challenges in the MIST sessions. Although there was no detailed data, the cancellation rate of the MIST sessions was not high. The simulation was scheduled at the same time every week, when the least elective clinical activities happened that facilitated attendance and avoided interruptions, with assigned providers and alternate staff to avoid non-attendance due to the increased patient volume or other unforeseen circumstances. To address concerns related to supply and equipment, many clinical materials such as endotracheal tubes, face masks, and umbilical catheter packages were reused for education after thorough cleaning and sterilization, making the training economically sustainable in developing countries. Of note, despite the effort of instructors and emphasis on the Vegas principle, the open training environment and large group size pose challenges to ensure the psychological safety of participants, especially the providers. We planned to perform quality improvement surveillance in the MIST program including the assurance of psychological safety.

### 4.2. Limitations

There are several limitations to this study. This is a retrospective before-after intervention study in a single center. The historical group is not an ideal control group. We were not able to distinguish other possible confounders which may have led to changes over time including the possible improvement in obstetric care, decreased birth rate, and the health of neonates. Collaboration between the obstetrical unit and NICU is important and involves regular joint departmental meetings and other interdisciplinary training, in addition to the weekly MIST. It is therefore unlikely to isolate the effects of simulation alone on neonatal outcomes. The Chinese definitions of neonatal asphyxia and severe asphyxia were different from those used internationally (25). Umbilical blood gas analysis was not available in some rural areas, so the diagnosis of a low Apgar score in this situation was also considered asphyxia in China. The diagnostic scheme for neonatal asphyxia recommended by the consensus is a dual-track system in China (24). Further, the incidence of MAS was based on the live births rather than neonates born through meconium-stained amniotic fluid because the number of neonates with meconium-stained amniotic fluid was not recorded. To improve and ensure the sustainability of MIST, teamwork assessment and objective post-simulation questionnaire including psychological safety should have been performed.

## 5. Conclusions

MIST on neonatal resuscitation is an effective training method to improve neonatal outcomes with decreased rates of neonatal asphyxia, severe asphyxia, HIE, and MAS. Implementation of regular and effective resuscitation training through the collaboration between departments promoted the development of neonatal resuscitation and improvement in patient safety. Additional well-designed, prospective research is needed to evaluate the effect of MIST on neonatal resuscitation in short- and long-term clinical outcomes.

## Data Availability

The raw data supporting the conclusions of this article will be made available by the authors, without undue reservation.
