# Supplementary material for: Improved neonatal outcomes by multidisciplinary simulation—a contemporary practice in the demonstration area of China
Source: Front Pediatr. 2023 Jun 8;11:1138633. doi: 10.3389/fped.2023.1138633 (PMC10287162; doi:10.3389/fped.2023.1138633)
Supplement: Supplementary file 1 [file Datasheet1.docx]

**Supplement: Corresponding tasks to different roles during team-based simulation**

|  | **Instructors** | **Providers** | **Designated Observers** |
| --- | --- | --- | --- |
| **Briefing** | ☑Brief introduction  ☑Set specific objectives  ☑Inform the participants about what will happen and what is expected from them during the simulation.  ☑Orientation: get participants to be aware of simulation environment, the simulator, the equipment and materials used during the simulation.* | ☑Anticipation (risk assessment)  ☑Plan (determine leadership role, role assignment, primary plan)  ☑Preparation (checklist)  ☑Discuss decisions  ☑Point out possible challenges | ☑Observe non-technical skills of participants. |
| **Simulation** | ☑Facilitate progression  ☑Create realism | ☑Perform resuscitation,  integration of multiple skills  ☑Call for help when needed | ☑Observe technical and non-technical skills of participants. |
| **Debriefing** | ☑Facilitate interactive discussion around objectives  ☑Listen to differing points and solve possible questions  ☑Combine scenarios with real life events, emphasis on patient outcome | ☑Expressed what was done well and need to be improved. | ☑Expressed what was done well and need to be improved. |
| **Conclusions** | Achieving the goals and objectives set originally | | |

*This is important to reduce interruption and improve smoothness prior to the team procedure.
